# Eight new species of the spider genus *Pimoa* (Araneae, Pimoidae) from Tibet, China

**DOI:** 10.3897/zookeys.940.49793

**Published:** 2020-06-11

**Authors:** Xiaoqing Zhang, Tianqi Lan, Lei Nie, Shuqiang Li

**Affiliations:** 1 Institute of Zoology, Chinese Academy of Sciences, Beijing 100101, China Institute of Zoology, Chinese Academy of Sciences Beijing China; 2 College of Life Science, Shenyang Normal University, Shenyang 110034, Liaoning, China Shenyang Normal University Liaoning China; 3 College of Life Science, Hebei University, Baoding, Hebei 071002, China Hebei University Baoding China

**Keywords:** Asia, description, diagnosis, taxonomy

## Abstract

Eight new species of the spider genus *Pimoa* Chamberlin & Ivie, 1943 are described from Tibet, China: *P. cona* Zhang & Li, **sp. nov.** (♂♀), *P. duiba* Zhang & Li, **sp. nov.** (♂♀), *P. lemenba* Zhang & Li, **sp. nov.** (♀), *P. mainling* Zhang & Li, **sp. nov.** (♂♀), *P. nyingchi* Zhang & Li, **sp. nov.** (♂♀), *P. rongxar* Zhang & Li, **sp. nov.** (♂♀), *P. samyai* Zhang & Li, **sp. nov.** (♂♀), and *P. yadong* Zhang & Li, **sp. nov.** (♂♀). The DNA barcodes of the eight new species are documented.

## Introduction

The spider family Pimoidae Wunderlich, 1986 contains 45 species in four genera: *Nanoa* Hormiga, Buckle & Scharff, 2005, *Pimoa* Chamberlin & Ivie, 1943, *Putaoa* Hormiga & Tu, 2008, and *Weintrauboa* Hormiga, 2003 (Li 2020). *Pimoa*, with the type species *Pimoa hespera* (Gertsch & Ivie, 1936) described from the USA, is the most species-rich genus in Pimoidae, with 33 described species prior to the current study. The genus is distributed on the west coast of the USA from Washington to California, the western Mediterranean (Alps and Cantabrian Mountains), and Asia (Himalaya to Beijing) (Mammola et al. 2016; Zhang and Li 2019; WSC 2020). Nine species are already known from China: *Pimoa anatolica* Hormiga, 1994, *P. binchuanensis* Zhang & Li, 2019, and *P. lihengae* Griswold, Long & Hormiga, 1999 from Yunnan Province; *P. lata* Xu & Li, 2009, *P. reniformis* Xu & Li, 2007, *P. trifurcata* Xu & Li, 2007, and *P. wanglangensis* Yuan, Zhao & Zhang, 2019 from Sichuan Province; *P. clavata* Xu & Li, 2007 from caves in the city of Beijing; and *P. xinjianensis* Zhang & Li, 2019 from three caves in Hunan Province (Griswold et al. 1999; Hormiga 1994a; Xu and Li 2007; Xu and Li 2009; Yuan et al. 2019; Zhang and Li 2019). In the present paper, eight new pimoids from Tibet, China are described.

## Materials and methods

Specimens were examined with a LEICA M205C stereomicroscope. Images were captured with an Olympus C7070 wide zoom digital camera (7.1 megapixels) mounted on an Olympus SZX12 dissecting microscope and assembled using Helicon Focus 3.10.3 image stacking software (Khmelik et al. 2006). Epigynes and male palps were examined after dissection from the spiders’ bodies. The left palps were illustrated unless otherwise noted. Epigynes were removed and treated in a warmed 10% potassium hydroxide (KOH) solution.

All measurements were obtained using a LEICA M205C stereomicroscope and are given in millimeters. We put legs and the body of the spider on the objective table of stereomicroscope and measured the length by a ruler in the eyepiece. Eye sizes were measured as the maximum diameter from either dorsal or frontal views. Leg measurements are shown as total length (femur, patella + tibia, metatarsus, tarsus). The terminology used in the text and the figure legends follows Hormiga (1994a). Distribution maps were generated using ArcView GIS (ESRI) software.

Abbreviations used in this paper and in the figure legends:

**ALE** anterior lateral eye;

**AME** anterior median eye;

**AME-ALE** distance between **AME** and **ALE**;

**AME-AME** distance between **AMEs**;

**AS** alveolar sclerite;

**C** conductor;

**CDP** cymbial denticulate process;

**CO** copulatory opening;

**DP** dorsal plate of the epigyne;

**E** embolus;

**FD** fertilization duct;

**MA** median apophysis;

**P** paracymbium;

**PCS** pimoid cymbial sclerite;

**PEP** pimoid embolic process;

**PLE** posterior lateral eye;

**PME** posterior median eye;

**PME-PLE** distance between **PME** and **PLE**;

**PME-PME** distance between **PMEs**;

**S** spermatheca;

**T** tegulum;

**VP** ventral plate of epigyne.

DNA barcodes were obtained for the eight new species by amplifying and sequencing a partial fragment of the mitochondrial gene cytochrome oxidase subunit I (COI) using primers LCO1490-oono (5’-CWACAAAYCATARRGATATTGG-3’) (Folmer et al. 1994; Miller et al. 2010) and HCO2198-zz (5’-TAAACTTCCAGGTGACCAAAAAATCA-3’) (Folmer et al. 1994; Zhao and Li 2017). For additional information on extraction, amplification, and sequencing procedures, see Wang et al. (2008). All sequences were checked for validity using BLAST and are deposited in GenBank. The accession numbers are provided in Table 1.

**Table 1.** Voucher specimen information.

Species	GenBank accession number	Sequence length	Collection locality
*Pimoa cona* sp. nov.	MT373707	654bp	Cona, Lhoka, Tibet, China
*Pimoa duiba* sp. nov.	MT373708	654bp	Duopozhang and Aza, Lhoka, Tibet, China
*Pimoa lemenba* sp. nov.	MT373706	654bp	Cona, Lhoka, Tibet, China
*Pimoa mainling* sp. nov.	MT373710	654bp	Mainling, Nyingchi, Tibet, China
*Pimoa nyingchi* sp. nov.	MT373713	654bp	Lulang, Nyingchi, Tibet, China
*Pimoa rongxar* sp. nov.	MT373712	654bp	Dinggyê, Shigatse, Tibet, China
*Pimoa samyai* sp. nov.	MT373711	654bp	Samyai Town, Lhoka, Tibet, China
*Pimoa yadong* sp. nov.	MT373709	654bp	Yadong, Shigatse, Tibet, China

All specimens (including molecular vouchers) are deposited in the Institute of Zoology, Chinese Academy of Sciences (**IZCAS**), Beijing, China.

## Taxonomy

**Family Pimoidae Wunderlich, 1986**

### Genus Pimoa

Taxon classificationAnimaliaAraneaePimoidae

Chamberlin & Ivie, 1943

123D03DE-3AF2-544D-A527-F83E8CB7B8CC

Pimoa : Chamberlin and Ivie 1943: 9; Hormiga 1994a: 4; Hormiga and Lew 2014: 1; Mammola et al. 2016: 1.

#### Type species.

*Labulla hespera* Gertsch & Ivie, 1936, from California, USA.

#### Diagnosis and description.

See Chamberlin and Ivie 1943; Gertsch and Ivie 1936; Griswold et al. 1998; Hormiga 1994a; Hormiga 1994b.

### Pimoa cona

Taxon classificationAnimaliaAraneaePimoidae

Zhang & Li, sp. nov .

7A028DD4-54C5-50A1-949C-44AC4ABAFCED

http://zoobank.org/B51D64B4-BBFD-4B42-AE2D-648926292E2E

Figures 1 , 2 , 16

#### Type material.

***Holotype***: ♂ (IZCAS-Ar40310), China, Tibet, Lhoka, Cona County, Senmuzha Scenic Area, 27.83°N, 91.73°E, elevation ca. 2845 m, 10.VIII.2018, X. Zhang and J. Liu leg. ***Paratypes***: 1♂2♀ (IZCAS-Ar40311-40313), same data as holotype; 1♂1♀ (IZCAS-Ar40314-40315), Cona County, Yelang Valley, 27.87°N, 91.81°E, elevation ca. 3379 m, 13.VIII.2018, X. Zhang and J. Liu leg.

#### Etymology.

The specific name is a noun in apposition taken from the type locality.

#### Diagnosis.

**Figure 1.**
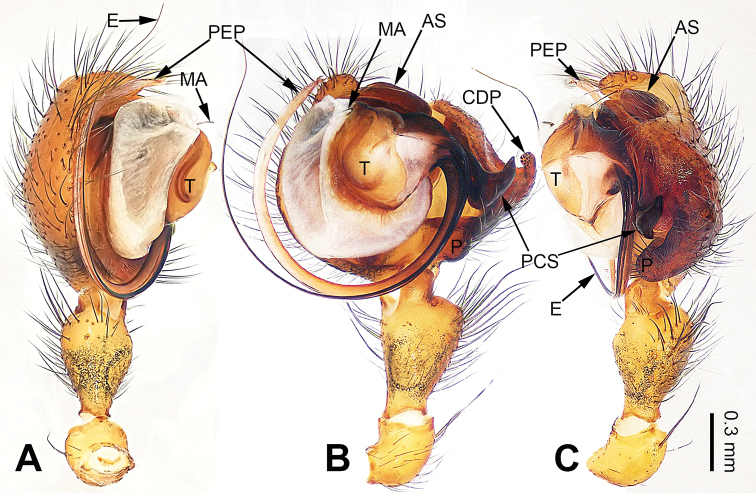
Left palp of *Pimoa cona* sp. nov., holotype **A** prolateral view **B** ventral view **C** retrolateral view. Abbreviations: AS = alveolar sclerite; CDP = cymbial denticulate process; E = embolus; MA = median apophysis; P = paracymbium; PCS = pimoid cymbial sclerite; PEP = pimoid embolic process; T = tegulum. Scale bar: equal for **A–C**.

**Figure 2.**
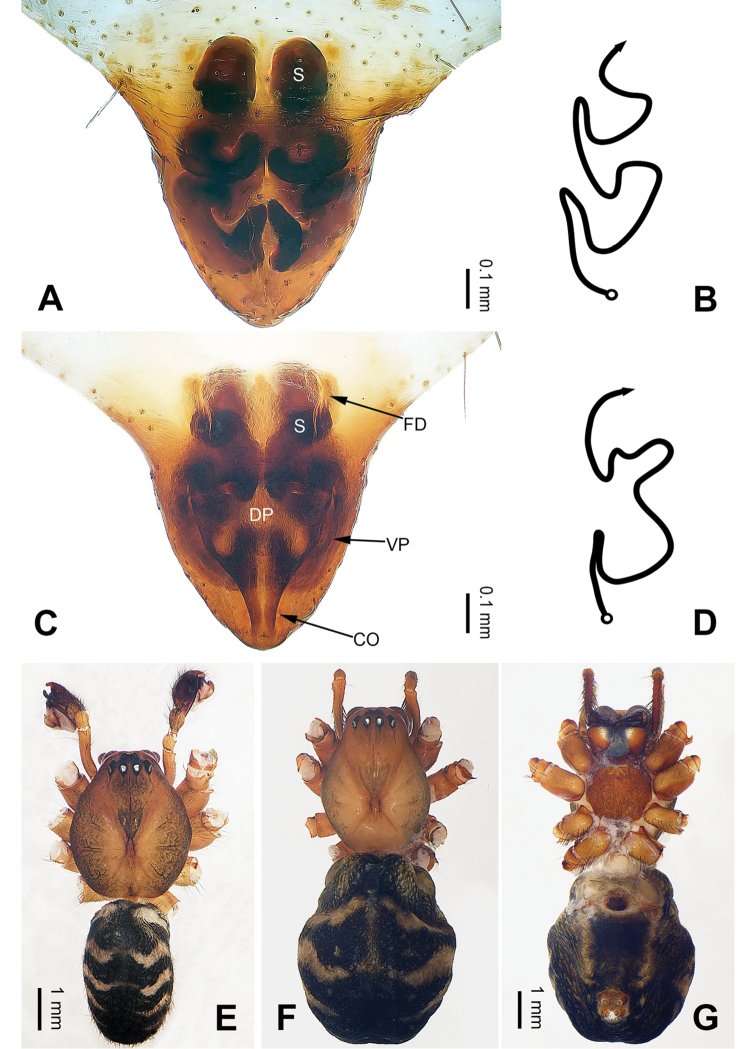
Epigyne and habitus of *Pimoa cona* sp. nov., female paratype and male holotype **A** epigyne, ventral view **B** schematic course of internal duct system, ventral view **C** vulva, dorsal view **D** schematic course of internal duct system, dorsal view **E** male habitus, dorsal view **F** female habitus, dorsal view **G** female habitus, ventral view. Abbreviations: CO = copulatory opening; DP = dorsal plate of the epigyne; FD = fertilization duct; S = spermatheca; VP = ventral plate of epigyne. Scale bars: equal for **F** and **G**.

The male of *Pimoa cona* sp. nov. resembles *P. nematoides* Hormiga, 1994 (see Hormiga 1994a: 71, figs 285–289) and *P. sinuosa* Hormiga, 1994 (see Hormiga 1994a: 67, figs 256–265) but can be distinguished by the large pimoid cymbial sclerite that is subdistally wide and distally pointed (Fig. 1B, vs. small and distally curved in *P. nematoides*; vs. slender and distally blunt in *P. sinuosa*); distinguished from *P. nematoides* by the long tibia, ca. 1/2 of the cymbial length (Fig. 1A–C, vs. short tibia, ca. 1/3 of cymbial length); distinguished from *P. sinuosa* by an embolus that begins at the 2:00 o’clock position (Fig. 1B, vs. an embolus that begins at the 5:30 o’clock position). The female of *P. cona* resembles *P. sinuosa* (see Hormiga 1994a: 67, figs 266–284) but can be distinguished by the pair of oval spermathecae (Fig. 2A, vs. subtriangular spermathecae) and by the subdistally narrow dorsal plate (Fig. 2C, vs. subdistally wide).

#### Description.

**Male** (**holotype**): Total length 7.24. Carapace 3.59 long, 2.95 wide. Abdomen 3.65 long, 2.18 wide. Eye sizes and interdistances: AME 0.18, ALE 0.20, PME 0.19, PLE 0.17; AME-AME 0.14, AME-ALE 0.17, PME-PME 0.16, PME-PLE 0.18. Leg measurements: I: 33.34 (9.36, 10.19, 9.94, 3.85); II: 32.52 (9.25, 10.13, 9.87, 3.27); III: 20.92 (6.16, 6.36, 6.35, 2.05); IV: – (7.95, –, –, –). Habitus as in Fig. 2E. Carapace brownish with black lateral margins; thoracic fovea and radial grooves distinct; sternum brownish. Abdomen black with yellowish transverse chevron bands. Legs brownish with black annulations, especially distinct on legs III and IV. Palp (Fig. 1A–C): patella short, ca. 1/2 of tibial length, with one retrolateral macroseta; tibia long, ca. 1/2 of cymbial length, with several macrosetae and a dorsal process; paracymbium short, ca. 1/3 of cymbial length, finger-shaped; pimoid cymbial sclerite large, subdistally wide and distally sharp, ca. 1/2 of cymbial length; cymbial denticulate process short and distally pointed, with more than 15 cuspules; median apophysis slender; conductor indistinct; pimoid embolic process with two short and sharp branches distally; embolus long and thin, longer than pimoid embolic process, beginning at the 2:00 o’clock position; embolic tooth absent.

**Female** (**paratype**): Total length 9.62. Carapace 3.72 long, 3.01 wide. Abdomen 5.90 long, 4.10 wide. Eye sizes and interdistances: AME 0.18, ALE 0.22, PME 0.19, PLE 0.21; AME-AME 0.14, AME-ALE 0.21, PME-PME 0.17, PME-PLE 0.20. Leg measurements: I: 27.83 (7.76, 9.17, 7.82, 3.08); II: 24.80 (6.79, 8.20, 6.99, 2.82); III: 17.56 (5.38, 5.51, 4.81, 1.86); IV: 22.31 (6.73, 7.37, 5.90, 2.31). Habitus as in Fig. 2F, G. Carapace brownish with black lateral margins; thoracic fovea and radial grooves distinct; sternum brownish. Abdomen black with yellowish transverse chevron bands and a short vertical band medially. Legs brownish with black annulations, especially distinct on legs III and IV. Epigyne (Fig. 2A–D): triangular; ventral plate broad, length subequal to width; dorsal plate narrow, with a blunt point; copulatory openings distinct; spermathecae oval, separated by ca. 1/3 width of spermatheca; fertilization ducts membranous, anteriorly oriented.

#### Distribution.

Known only from the type locality, Tibet, China (Fig. 16).

### Pimoa duiba

Taxon classificationAnimaliaAraneaePimoidae

Zhang & Li, sp. nov .

95D5916B-50C2-5B67-975C-AE6E71C9F25D

http://zoobank.org/2A93ED2B-AFAE-4EB6-B1BE-ACB9752BBF9D

Figures 3 , 4 , 16

#### Type material.

***Holotype***: ♂ (IZCAS-Ar40316), China, Tibet, Lhoka, Duopozhang Town, Duiba Village, 29.37°N, 91.70°E, elevation ca. 4095 m, 14.VIII.2019, X. Zhang, Z. Bai and J. Liu leg. ***Paratypes***: 1♀ (IZCAS-Ar40317), same data as holotype; 1♀ (IZCAS-Ar40318), Lhoka, Aza Town, Beside the stream behind Zonggongbu Cave, 29.37°N, 91.32°E, elevation ca. 4537 m, 29.VIII.2018, X. Zhang and J. Liu leg.

#### Etymology.

The specific name is a noun in apposition taken from the type locality.

#### Diagnosis.

**Figure 3.**
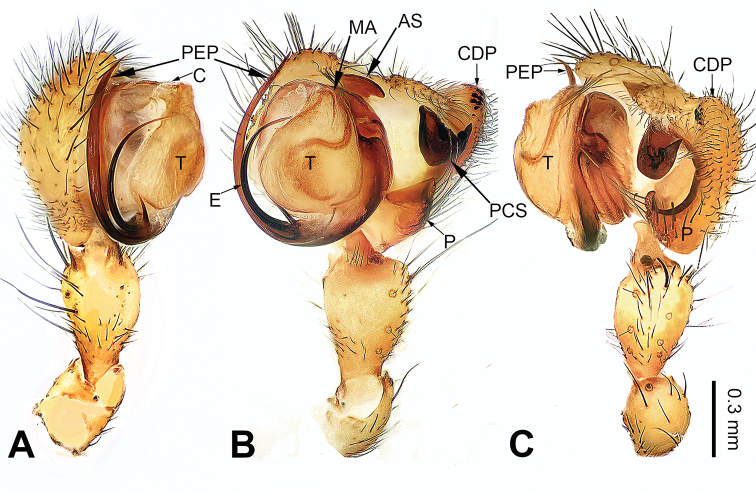
Left palp of *Pimoa duiba* sp. nov., holotype **A** prolateral view **B** ventral view **C** retrolateral view. Abbreviations: AS = alveolar sclerite; C = conductor; CDP = cymbial denticulate process; E = embolus; MA = median apophysis; P = paracymbium; PCS = pimoid cymbial sclerite; PEP = pimoid embolic process; T = tegulum. Scale bar: equal for **A–C**.

**Figure 4.**
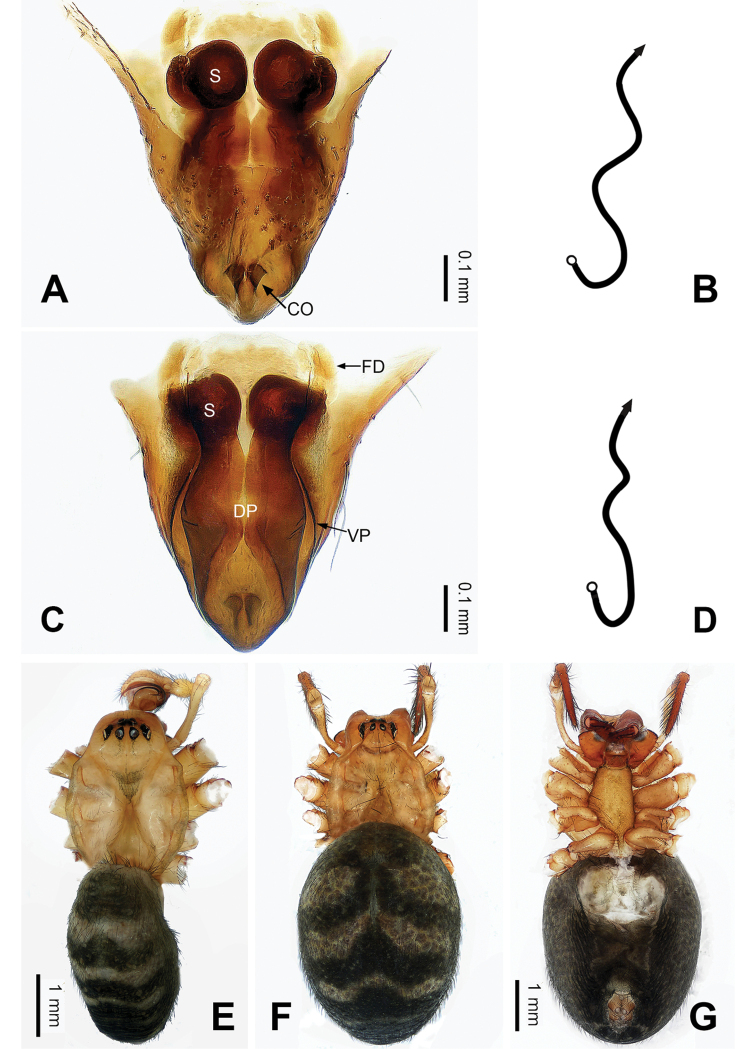
Epigyne and habitus of *Pimoa duiba* sp. nov., female paratype and male holotype **A** epigyne, ventral view **B** schematic course of internal duct system, ventral view **C** vulva, dorsal view **D** schematic course of internal duct system, dorsal view **E** male habitus, dorsal view **F** female habitus, dorsal view **G** female habitus, ventral view. Abbreviations: CO = copulatory opening; DP = dorsal plate of the epigyne; FD = fertilization duct; S = spermatheca; VP = ventral plate of epigyne. Scale bars: equal for **F** and **G**.

The male of *Pimoa duiba* sp. nov. resembles *P. samyai* sp. nov. (Fig. 12A–C) and *P. trifurcata* (see Xu and Li 2007: 496, figs 48–54) but can be distinguished by the short and distally blunt cymbial denticulate process (Fig. 3B, vs. relatively long and distally narrow in *P. samyai* and *P. trifurcata*); distinguished from *P. samyai* by the nearly V-shaped pimoid cymbial sclerite (Fig. 3B, vs. nearly L-shaped); distinguished from *P. trifurcata* by the pimoid embolic process without a trifurcate apex (Fig. 3A–C, vs. with a trifurcate apex). The female of *P. duiba* also resembles *P. samyai* sp. nov. (Fig. 13A–D) and *P. trifurcata* (see Xu and Li 2007: 496, figs 55–61) but can be distinguished by the short distance between the spermathecae (Fig. 4A, vs. separated by ca. 1/2 the width of a spermatheca in *P. samyai* and *P. trifurcata*); distinguished from *P. samyai* by having a spermatheca that is wider than long (Fig. 4A, vs. longer than wide); distinguished from *P. trifurcata* by the medially narrow dorsal plate (Fig. 4C, vs. medially relatively wide).

#### Description.

**Male** (**holotype**): Total length 5.26. Carapace 2.18 long, 1.86 wide. Abdomen 3.08 long, 1.73 wide. Eye sizes and interdistances: AME 0.12, ALE 0.13, PME 0.12, PLE 0.12; AME-AME 0.07, AME-ALE 0.11, PME-PME 0.10, PME-PLE 0.14. Leg measurements: I: 23.84 (6.60, 7.95, 6.73, 2.56); II: 21.79 (5.90, 7.24, 6.34, 2.31); III: 16.16 (4.62, 5.45, 4.49, 1.60); IV: 19.41 (5.51, 6.15, 5.83, 1.92). Promargin of chelicerae with three teeth, retromargin with two teeth. Habitus as in Fig. 4E. Carapace yellowish with slightly darker lateral margins; thoracic fovea and radial grooves distinct; sternum brownish. Abdomen black with yellowish transverse chevron bands. Legs brownish without black annulations. Palp (Fig. 3A–C): patella short, ca. 1/2 of tibial length, with a single retrolateral macroseta; tibia long, ca. 1/2 of cymbial length, with several macrosetae and a dorsal process; paracymbium short, ca. 1/3 of cymbial length, hook-shaped; pimoid cymbial sclerite nearly V-shaped, ca. 1/3 of cymbial length; cymbial denticulate process short and distally blunt, with more than five cuspules; median apophysis slender; conductor distinct; pimoid embolic process distally pointed, longer than embolus; embolus beginning at the 7:00 o’clock position, with short slender spine proximally; embolic tooth absent.

**Female** (**paratype**): Total length 6.85. Carapace 2.56 long, 2.11 wide. Abdomen 4.29 long, 3.27 wide. Eye sizes and interdistances: AME 0.12, ALE 0.16, PME 0.10, PLE 0.15; AME-AME 0.09, AME-ALE 0.15, PME-PME 0.09, PME-PLE 0.17. Leg measurements: I: 21.47 (6.15, 6.73, 6.03, 2.56); II: 19.55 (5.38, 6.67, 5.38, 2.12); III: 15.20 (4.62, 4.81, 4.10, 1.67); IV: 19.23 (5.51, 6.22, 5.45, 2.05). Promargin of chelicerae with three teeth, retromargin with two teeth. Habitus as in Fig. 4F, G. Carapace brownish with slightly darker lateral margins; thoracic fovea and radial grooves indistinct; sternum brownish. Abdomen black with yellowish transverse chevron bands. Legs brownish without black annulations. Epigyne (Fig. 4A–D): triangular; ventral plate broad, length subequal to width; dorsal plate longer than wide, nearly tongue-shaped; copulatory openings distinct; spermathecae nearly round with short distance between them; fertilization ducts yellowish, anteriorly oriented.

#### Distribution.

Known only from the type locality, Tibet, China (Fig. 16).

### Pimoa lemenba

Taxon classificationAnimaliaAraneaePimoidae

Zhang & Li, sp. nov .

2F9304D1-D770-5857-9613-38010DD30A77

http://zoobank.org/643BA218-A6BB-4CC1-AEA5-60CD91E84F5D

Figures 5 , 16

#### Type material.

***Holotype***: ♀ (IZCAS-Ar40319), China, Tibet, Lhoka, Cona County, Lemenba Town, 17–20 km section from Lewang Bridge to Liulian Highway, 27.80°N, 91.77°E, elevation ca. 3706 m, 5.VI.2016, J. Wu leg.

#### Etymology.

The specific name is a noun in apposition taken from the type locality.

#### Diagnosis.

**Figure 5.**
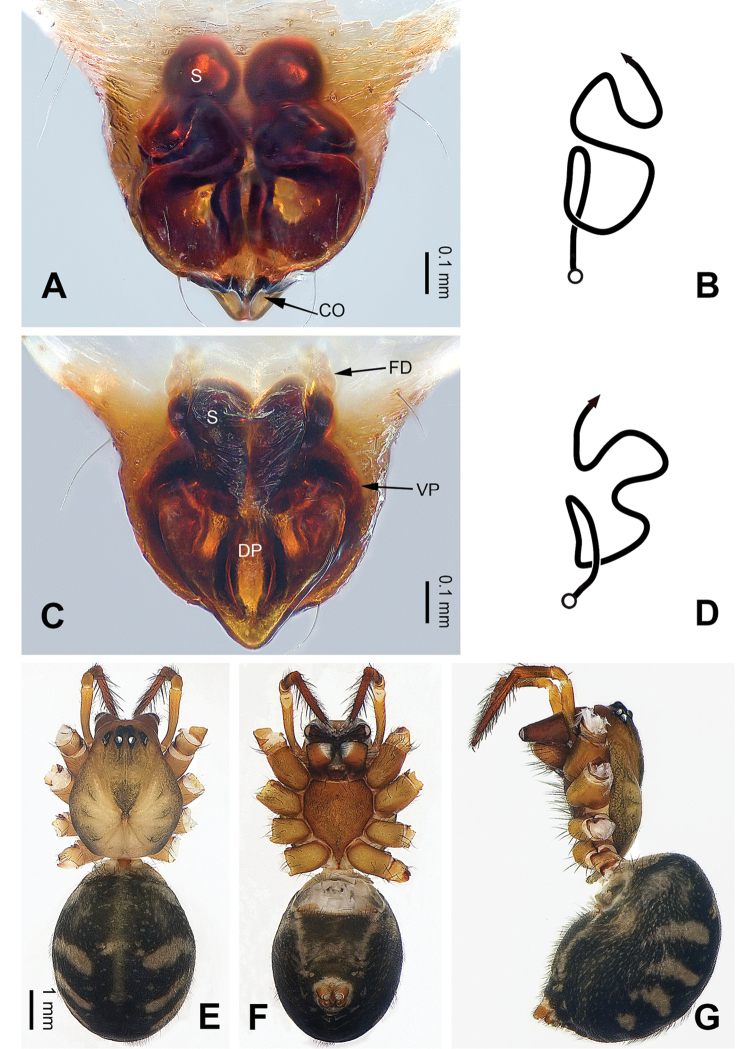
Epigyne and habitus of *Pimoa lemenba* sp. nov., female holotype **A** epigyne, ventral view **B** schematic course of internal duct system, ventral view **C** vulva, dorsal view **D** schematic course of internal duct system, dorsal view **E** female habitus, dorsal view **F** female habitus, ventral view **G** female habitus, lateral view. Abbreviations: CO = copulatory opening; DP = dorsal plate of the epigyne; FD = fertilization duct; S = spermatheca; VP = ventral plate of epigyne. Scale bars: equal for **E–G**.

The species resembles *Pimoa sinuosa* Hormiga, 1994 (see Hormiga 1994a: 67, figs 266–284) but can be distinguished by the pair of round spermathecae which are close together (Fig. 5A, vs. separated by ca. 1/2 the width of a spermatheca), by the medially wide dorsal plate (Fig. 5C, vs. medially relatively narrow), and by the abdomen with the vertical band not extending to the distal part (Fig. 5E, vs. vertical band absent).

#### Description.

**Female** (**holotype**): Total length 8.59. Carapace 3.40 long, 2.88 wide. Abdomen 5.19 long, 3.46 wide. Eye sizes and interdistances: AME 0.17, ALE 0.20, PME 0.19, PLE 0.18; AME-AME 0.10, AME-ALE 0.19, PME-PME 0.14, PME-PLE 0.22. Leg measurements: I: – (7.12, –, –, –); II: – (6.47, –, –, –); III: missing; IV: missing. Habitus as in Fig. 5E–G. Carapace yellowish with black lateral margins; thoracic fovea and radial grooves distinct; sternum brownish. Abdomen black with yellowish transverse bands and a vertical band not extending to distal part. Legs brownish with black annulations. Epigyne (Fig. 5A–D): ventral and dorsal plates broad, length subequal to width; copulatory openings distinct; spermathecae round, close to each other; fertilization ducts crystalline, anteriorly oriented.

**Male**: unknown.

#### Distribution.

Known only from the type locality, Tibet, China (Fig. 16).

### Pimoa mainling

Taxon classificationAnimaliaAraneaePimoidae

Zhang & Li, sp. nov .

32AF296A-4146-5023-A4D0-703BD62C056B

http://zoobank.org/75899015-5478-4F26-AB92-E96010E8185D

Figures 6 , 7 , 16

#### Type material.

***Holotype***: ♂ (IZCAS-Ar40320), China, Tibet, Nyingchi, Mainling County, along the way from Zhagonggou Scenic Area to Ganlu Cave, 29.16°N, 94.23°E, elevation ca. 3440 m, 27.VIII.2018, X. Zhang and J. Liu leg. ***Paratypes***: 2♀ (IZCAS-Ar40321-Ar40322), same data as holotype.

#### Etymology.

The specific name is a noun in apposition taken from the type locality.

#### Diagnosis.

**Figure 6.**
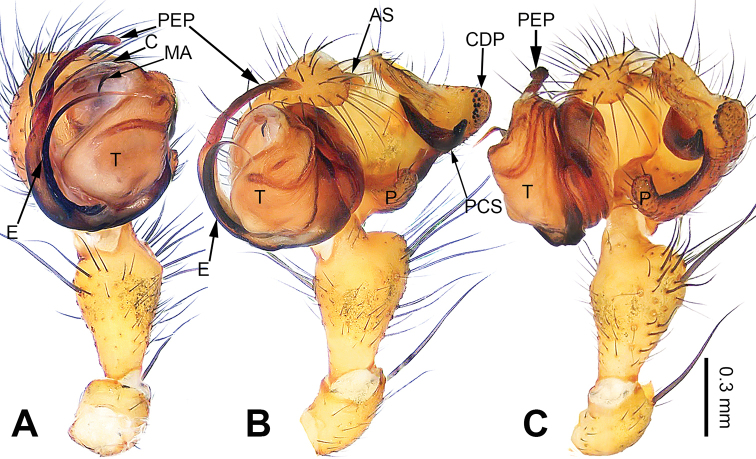
Left palp of *Pimoa mainling* sp. nov., holotype **A** prolateral view **B** ventral view **C** retrolateral view. Abbreviations: AS = alveolar sclerite; C = conductor; CDP = cymbial denticulate process; E = embolus; MA = median apophysis; P = paracymbium; PCS = pimoid cymbial sclerite; PEP = pimoid embolic process; T = tegulum. Scale bar: equal for **A–C**.

**Figure 7.**
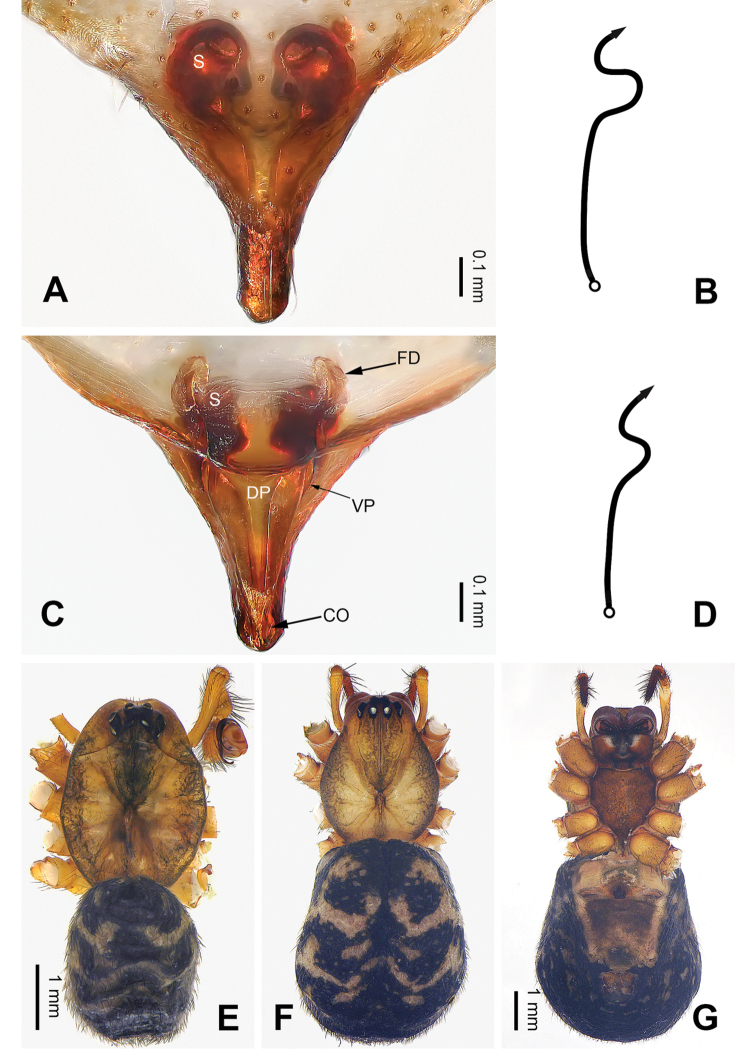
Epigyne and habitus of *Pimoa mainling* sp. nov., female paratype and male holotype **A** epigyne, ventral view **B** schematic course of internal duct system, ventral view **C** vulva, dorsal view **D** schematic course of internal duct system, dorsal view **E** male habitus, dorsal view **F** female habitus, dorsal view **G** female habitus, ventral view. Abbreviations: CO = copulatory opening; DP = dorsal plate of the epigyne; FD = fertilization duct; S = spermatheca; VP = ventral plate of epigyne. Scale bars: equal for **F** and **G**.

The male of *Pimoa mainling* sp. nov. resembles *P. binchuanensis* (see Zhang and Li 2019: 3, figs 1, 2) but can be distinguished by the distally curved and nearly hook-shaped pimoid cymbial sclerite (Fig. 6B, vs. medially curved and nearly U-shaped). The male of *P. mainling* also resembles *P. crispa* Hormiga, 1994 (see Hormiga 1994a: 63, figs 233–238; Hormiga 1994b: fig 1A, B) but can be distinguished by a distally narrow cymbial denticulate process with few cuspules (Fig. 6B, vs. distally wide cymbial denticulate process with many cuspules). The female of *P. mainling* resembles *P. crispa* Hormiga, 1994 (see Hormiga 1994a: 63, figs 239–247) but can be distinguished by the distance between the pair of spermathecae which is ca. 1/3 the width of a spermatheca (Fig. 7A, vs. shorter distance between spermathecae) and by the funnel-shaped epigyne, which is distally straight and long (Fig. 7A–D, vs. triangular epigyne).

#### Description.

**Male** (**holotype**): Total length 5.06. Carapace 2.56 long, 2.18 wide. Abdomen 2.50 long, 2.05 wide. Eye sizes and interdistances: AME 0.16, ALE 0.17, PME 0.13, PLE 0.17; AME-AME 0.13, AME-ALE 0.17, PME-PME 0.10, PME-PLE 0.16. Leg measurements: I: 25.38 (6.99, 8.40, 7.49, 2.50); II: – (5.90, –, –, –); III: 13.52 (4.17, 4.23, 3.65, 1.47); IV: 16.79 (5.19, 5.38, 4.55, 1.67). Habitus as in Fig. 7E. Carapace brownish with black lateral margins; thoracic fovea and radial grooves distinct; sternum brownish. Abdomen black with yellowish transverse chevron bands. Legs brownish with black annulations, especially distinct on legs III and IV. Palp (Fig. 6A–C): patella short, ca. 1/3 of tibial length, with one retrolateral macroseta; tibia almost the same length as cymbium, with several macrosetae and a dorsal process; paracymbium short, ca. 1/3 of cymbial length, hook-shaped; pimoid cymbial sclerite distally curved, ca. 1/2 of cymbial length; cymbial denticulate process short, distally narrow and blunt, with more than ten cuspules; median apophysis slender; conductor distinct; pimoid embolic process long, slightly wider distally; embolus beginning at the 7:30 o’clock position; embolic tooth absent.

**Female** (**paratype**): Total length 8.78. Carapace 3.40 long, 3.01 wide. Abdomen 5.38 long, 4.55 wide. Eye sizes and interdistances: AME 0.17, ALE 0.19, PME 0.19, PLE 0.20; AME-AME 0.15, AME-ALE 0.17, PME-PME 0.17, PME-PLE 0.21. Leg measurements: I: 21.22 (6.15, 7.37, 5.58, 2.12); II: 18.33 (5.32, 6.22, 4.74, 2.05); III: 13.14 (4.17, 4.10, 3.40, 1.47); IV: – (5.06, 5.51, 4.42, –). Habitus as in Fig. 7F, G. Carapace yellowish with black lateral margins; thoracic fovea and radial grooves distinct; sternum brownish. Abdomen black with yellowish transverse chevron bands. Legs brownish with distinct black annulations on all legs. Epigyne (Fig. 7A–D): funnel-shaped; ventral and dorsal plates narrow; copulatory openings distinct; spermathecae nearly oval, separated by ca. 1/3 width of spermatheca; fertilization ducts membranous, anteriorly oriented.

#### Distribution.

Known only from the type locality, Tibet, China (Fig. 16).

### Pimoa nyingchi

Taxon classificationAnimaliaAraneaePimoidae

Zhang & Li, sp. nov .

9796C136-1D75-5159-874C-2D0F5D0C4F4C

http://zoobank.org/AA082852-47BB-4A5B-AF4D-78E06176DDC6

Figures 8 , 9 , 16

#### Type material.

***Holotype***: ♂ (IZCAS-Ar40323), China, Tibet, Nyingchi, near Lunang Town, 29.94°N, 94.80°E, elevation ca. 2615 m, 25.VIII.2018, X. Zhang and J. Liu leg. ***Paratypes***: 1♂2♀ (IZCAS-Ar40324-Ar40326), same data as holotype; 2♂2♀ (IZCAS-Ar40327-Ar40330), Nyingchi, Near Sejila Pass, 29.56°N, 94.57°E, elevation ca. 3764 m, 26.VIII.2018, X. Zhang and J. Liu leg.

#### Etymology.

The specific name is a noun in apposition taken from the type locality.

#### Diagnosis.

**Figure 8.**
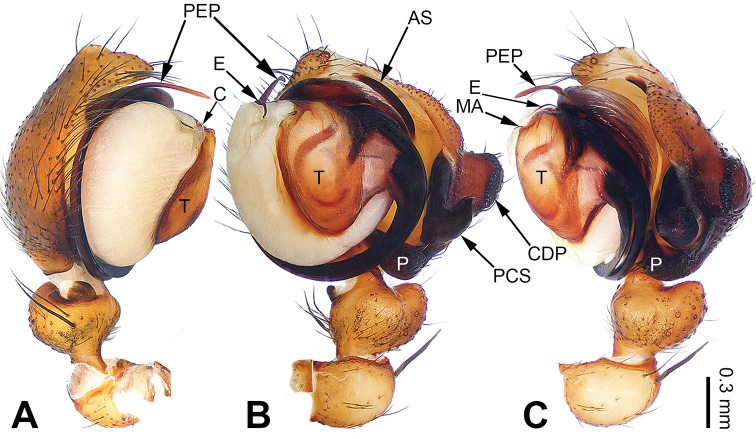
Left palp of *Pimoa nyingchi* sp. nov., holotype **A** prolateral view **B** ventral view **C** retrolateral view. Abbreviations: AS = alveolar sclerite; C = conductor; CDP = cymbial denticulate process; E = embolus; MA = median apophysis; P = paracymbium; PCS = pimoid cymbial sclerite; PEP = pimoid embolic process; T = tegulum. Scale bar: equal for **A–C.**

**Figure 9.**
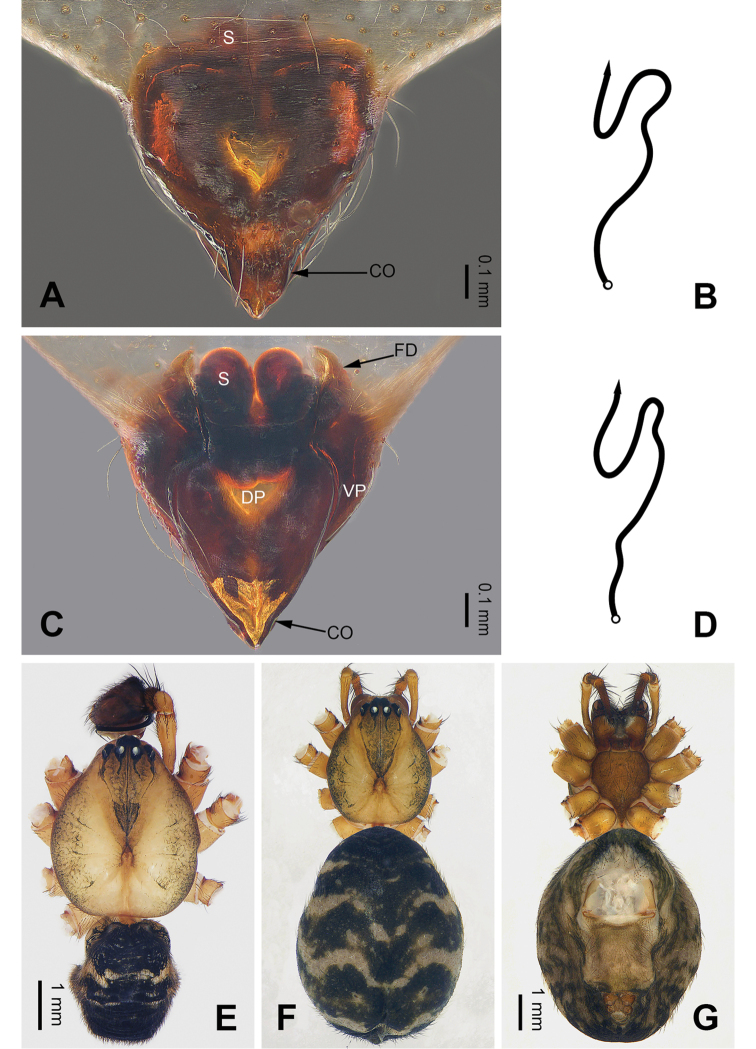
Epigyne and habitus of *Pimoa nyingchi* sp. nov., female paratype and male holotype **A** epigyne, ventral view **B** schematic course of internal duct system, ventral view **C** vulva, dorsal view **D** schematic course of internal duct system, dorsal view **E** male habitus, dorsal view **F** female habitus, dorsal view **G** female habitus, ventral view. Abbreviations: CO = copulatory opening; DP = dorsal plate of the epigyne; FD = fertilization duct; S = spermatheca; VP = ventral plate of epigyne. Scale bars: equal for **F** and **G**.

The male of *Pimoa nyingchi* sp. nov. resembles *P. reniformis* (see Xu and Li 2007: 493, figs 36–41) but can be distinguished by the long, distally flat and wide pimoid cymbial sclerite (Fig. 8B, vs. narrow and distally curved) and by the relatively large and wide paracymbium (Fig. 8B, C, vs. small and narrow). The female of *P. nyingchi* also resembles *P. reniformis* (see Xu and Li 2007: 493, figs 42–47) but can be distinguished by a pair of small, oval spermathecae (Fig. 9C, vs. large and kidney-shaped) and by the broad dorsal plate (Fig. 9C, vs. narrow dorsal plate).

#### Description.

**Male** (**holotype**): Total length 7.05. Carapace 3.59 long, 3.01 wide. Abdomen 3.46 long, 2.44 wide. Eye sizes and interdistances: AME 0.17, ALE 0.17, PME 0.18, PLE 0.16; AME-AME 0.14, AME-ALE 0.16, PME-PME 0.15, PME-PLE 0.18. Leg measurements: I: 28.08 (7.63, 8.91, 8.01, 3.53); II: 16.92 (5.89, 5.26, 3.78, 1.99); III: 16.73 (5.00, 5.19, 4.62, 1.92); IV: 14.10 (5.45, 4.23, 2.95, 1.47). Habitus as in Fig. 9E. Carapace yellowish with black lateral margins; thoracic fovea and radial grooves distinct; sternum brownish. Abdomen black with yellowish transverse bands. Legs brownish with distinct black annulations on all legs. Palp (Fig. 8A–C): patella short, almost the same length as tibia, with one retrolateral macroseta; tibia short, ca. 1/3 of cymbial length, with several macrosetae and a dorsal process; paracymbium short, ca. 1/3 of cymbial length, hook-shaped; pimoid cymbial sclerite long, distally flat and wide, ca. 1/2 of cymbial length; cymbial denticulate process short, distally wide and bent inward, with more than 20 cuspules; median apophysis slender; conductor distinct; pimoid embolic process almost the same length as embolus; embolus beginning at the 3:00 o’clock position; embolic tooth absent.

**Female** (**paratype**): Total length 9.49. Carapace 3.27 long, 2.88 wide. Abdomen 6.22 long, 4.68 wide. Eye sizes and interdistances: AME 0.19, ALE 0.20, PME 0.19, PLE 0.20; AME-AME 0.14, AME-ALE 0.12, PME-PME 0.15, PME-PLE 0.17. Leg measurements: I: 20.76 (6.09, 6.79, 5.38, 2.50); II: 17.49 (5.06, 5.89, 4.68, 1.86); III: – (3.97, –, –, –); IV: 16.34 (5.06, 5.58, 4.42, 1.28). Habitus as in Fig. 9F, G. Carapace yellowish with black lateral margins; thoracic fovea and radial grooves distinct; sternum brownish. Abdomen black with yellowish transverse chevron bands. Legs brownish with distinct black annulations on all legs. Epigyne (Fig. 9A–D): triangular; ventral plate broad, length subequal to width; dorsal plate wide medially and pointed distally; copulatory openings indistinct; spermathecae oval, close to each other; fertilization ducts laterally oriented.

#### Distribution.

Known only from the type locality, Tibet, China (Fig. 16).

### Pimoa rongxar

Taxon classificationAnimaliaAraneaePimoidae

Zhang & Li, sp. nov .

E6BBC56D-6580-54D9-8A68-44E4A68465E5

http://zoobank.org/F5A97419-F64D-4B8C-ACFD-D88831A6CABA

**Figure 10.**
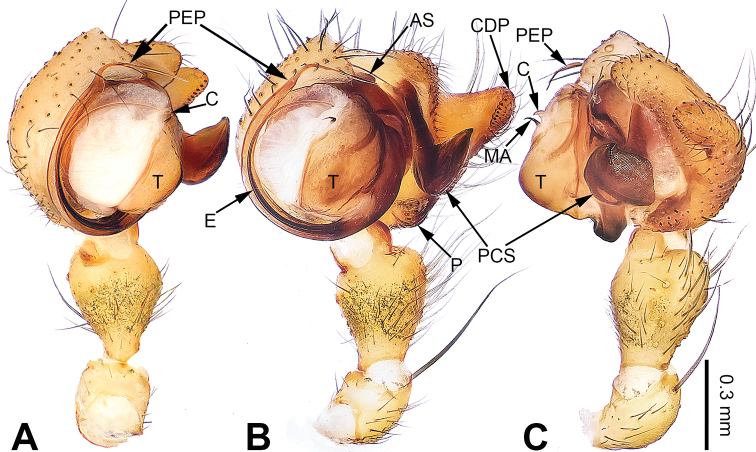
Left palp of *Pimoa rongxar* sp. nov., holotype **A** prolateral view **B** ventral view **C** retrolateral view. Abbreviations: AS = alveolar sclerite; C = conductor; CDP = cymbial denticulate process; E = embolus; MA = median apophysis; P = paracymbium; PCS = pimoid cymbial sclerite; PEP = pimoid embolic process; T = tegulum. Scale bar: equal for **A–C**.

**Figure 11.**
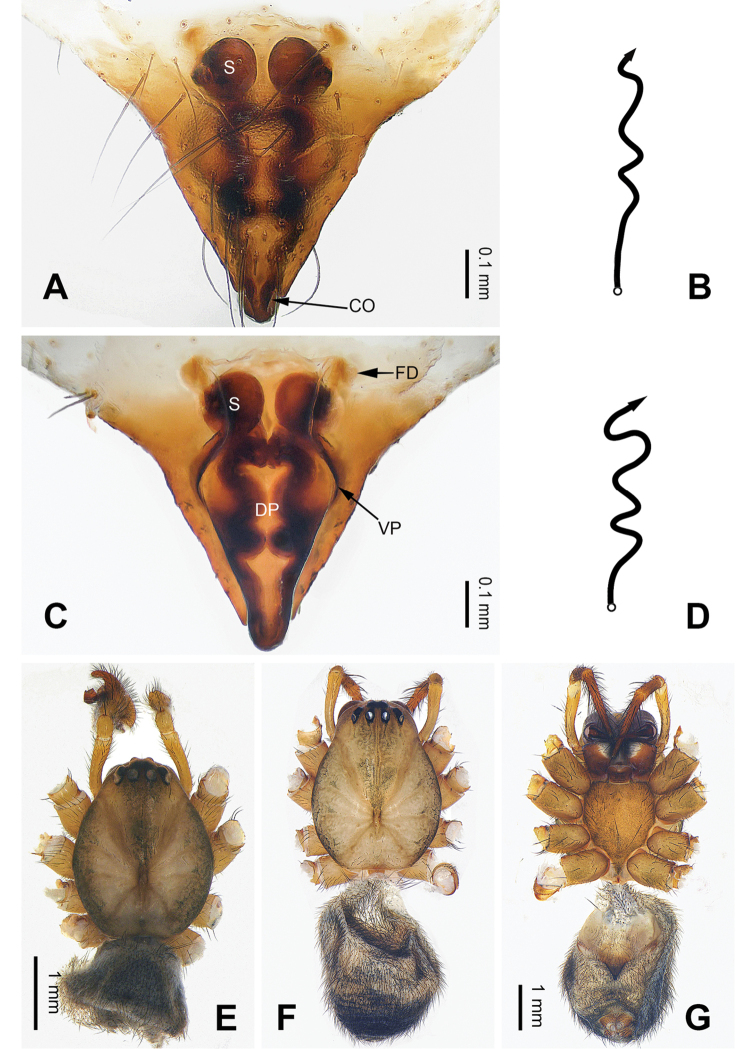
Epigyne and habitus of *Pimoa rongxar* sp. nov., female paratype and male holotype **A** epigyne, ventral view **B** schematic course of internal duct system, ventral view **C** vulva, dorsal view **D** schematic course of internal duct system, dorsal view **E** male habitus, dorsal view **F** female habitus, dorsal view **G** female habitus, ventral view. Abbreviations: CO = copulatory opening; DP = dorsal plate of the epigyne; FD = fertilization duct; S = spermatheca; VP = ventral plate of epigyne. Scale bars: equal for **F** and **G**.

Figures 10 , 11 , 16

#### Type material.

***Holotype***: ♂ (IZCAS-Ar40331), China, Tibet, Shigatse, Dinggyê County, Rongxar Town, Woods by the river, 28.07°N, 86.37°E , elevation ca. 3520 m, 29.VII.2018, X. Zhang and J. Liu leg. ***Paratype***: 1♀ (IZCAS-Ar40332), same data as holotype.

#### Etymology.

The specific name is a noun in apposition taken from the type locality.

#### Diagnosis.

The male of *Pimoa rongxar* sp. nov. resembles *P. reniformis* (see Xu and Li 2007: 493, figs 36–41) and *P. thaleri* Trotta, 2009 (see Trotta 2009: 1404, fig. 1) but can be distinguished by the large, long and subdistally wide pimoid cymbial sclerite (Fig. 10B, vs. small and narrow in *P. reniformis*; vs. short and medially wide in *P. thaleri*); distinguished from *P. reniformis* by the long palpal tibia, ca. 1/2 of the cymbial length (Fig. 10A–C, vs. palpal tibia short, ca. 1/3 of the cymbial length); distinguished from *P. thaleri* by the pimoid embolic process which is longer than the embolus (Fig. 10B, vs. a pimoid embolic process that is almost the same length as the embolus). The female of *P. rongxar* resembles *P. indiscreta* Hormiga, 1994 (see Hormiga 1994a: 66, figs 248–255) but can be distinguished by a pair of nearly round spermathecae (Fig. 11A, vs. nearly oval) and by the laterally oriented pair of fertilization ducts (Fig. 11A–D, vs. medially oriented fertilization ducts).

#### Description.

**Male** (**holotype**): Total length 3.97. Carapace 2.50 long, 1.92 wide. Abdomen 1.47 long, 1.86 wide. Eye sizes and interdistances: AME 0.12, ALE 0.14, PME 0.16, PLE 0.14; AME-AME 0.14, AME-ALE 0.15, PME-PME 0.11, PME-PLE 0.11. Leg measurements: I: 19.23 (5.26, 6.22, 5.19, 2.56); II: 16.79 (4.55, 5.38, 4.62, 2.24); III: 11.48 (3.27, 3.46, 3.21, 1.54); IV: 14.16 (3.97, 4.55, 4.04, 1.60). Habitus as in Fig. 11E. Carapace brownish with black lateral margins; thoracic fovea and radial grooves distinct; sternum brownish. Abdomen dark gray. Legs brownish with distinct black annulations on all legs. Palp (Fig. 10A–C): patella short, ca. 1/2 of tibial length, with a single retrolateral macroseta; tibia long, ca. 1/2 of cymbial length, with several macrosetae and a dorsal process; paracymbium short, ca. 1/3 of cymbial length; pimoid cymbial sclerite large, long and subdistally wide, slightly shorter than cymbial length; cymbial denticulate process short, distally narrow, with more than ten cuspules; median apophysis slender; conductor distinct; pimoid embolic process longer than embolus, abruptly narrowing; embolus beginning at the 5:30 o’clock position; embolic tooth absent.

**Female** (**paratype**): Total length 7.63. Carapace 3.78 long, 2.95 wide. Abdomen 3.85 long, 2.63 wide. Eye sizes and interdistances: AME 0.17, ALE 0.20, PME 0.18, PLE 0.17; AME-AME 0.13, AME-ALE 0.17, PME-PME 0.18, PME-PLE 0.21. Leg measurements: I: 24.29 (6.67, 8.14, 6.60, 2.88); II: 22.50 (6.35, 7.44, 6.15, 2.56); III: 17.38 (5.19, 5.58, 4.62, 1.99); IV: 20.77 (6.15, 6.86, 5.58, 2.18). Habitus as in Fig. 11F, G. Carapace yellowish with black lateral margins; thoracic fovea and radial grooves distinct; sternum brownish. Abdomen yellowish with black marks. Legs yellowish with distinct black annulations on all legs. Epigyne (Fig. 11A–D): triangular; ventral plate broad, length subequal to width; dorsal plate narrow, longer than wide; copulatory openings distinct; spermathecae nearly round, separated by ca. 1/4 width of spermatheca; fertilization ducts yellowish, laterally oriented.

#### Distribution.

Known only from the type locality, Tibet, China (Fig. 16).

### Pimoa samyai

Taxon classificationAnimaliaAraneaePimoidae

Zhang & Li, sp. nov .

882578C9-D067-5732-9A45-B74E7C4CE999

http://zoobank.org/F6AADDC6-7CDA-4DD3-B406-D028D22913A0

**Figure 12.**
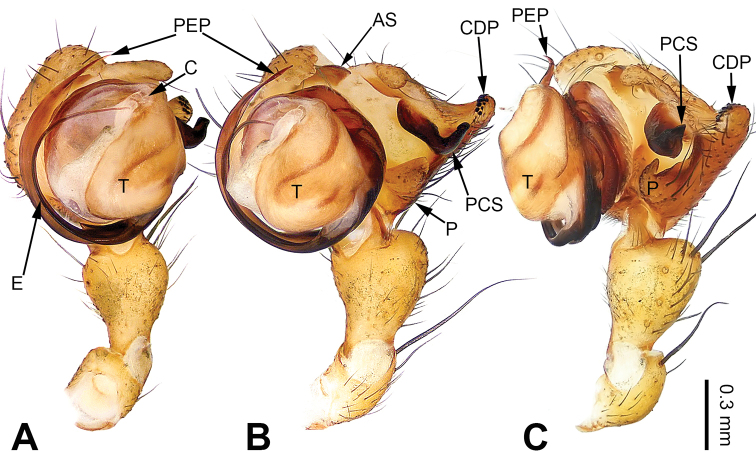
Left palp of *Pimoa samyai* sp. nov., holotype **A** prolateral view **B** ventral view **C** retrolateral view. Abbreviations: AS = alveolar sclerite; C = conductor; CDP = cymbial denticulate process; E = embolus; P = paracymbium; PCS = pimoid cymbial sclerite; PEP = pimoid embolic process; T = tegulum. Scale bar: equal for **A–C**.

**Figure 13.**
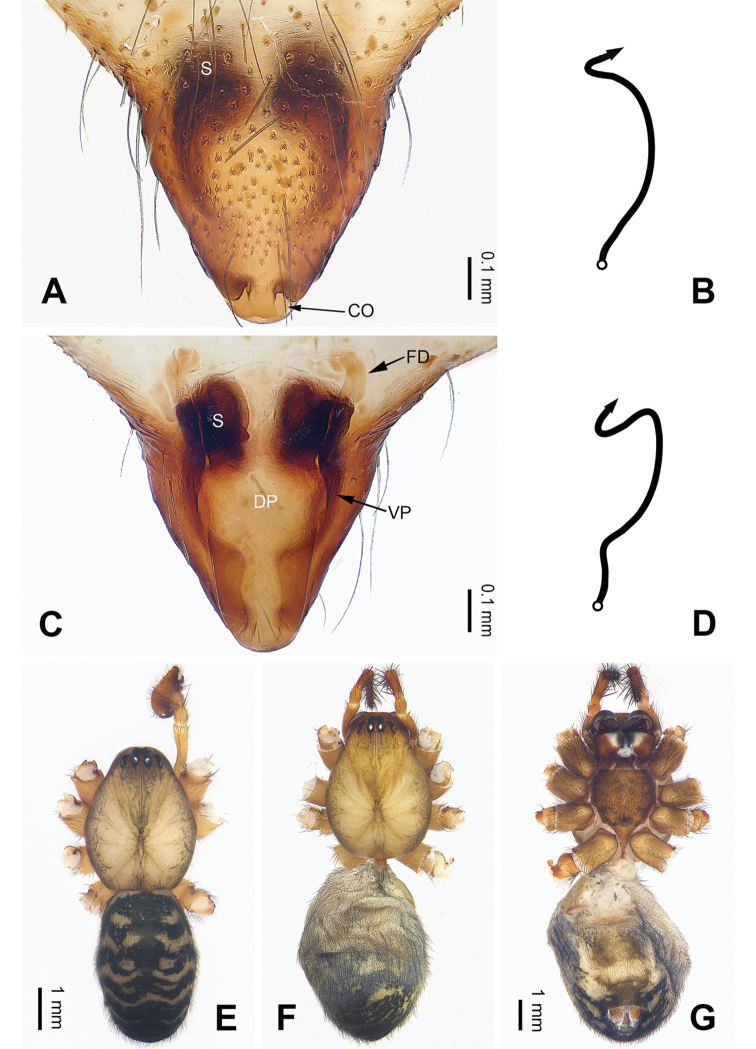
Epigyne and habitus of *Pimoa samyai* sp. nov., female paratype and male holotype **A** epigyne, ventral view **B** schematic course of internal duct system, ventral view **C** vulva, dorsal view **D** schematic course of internal duct system, dorsal view **E** male habitus, dorsal view **F** female habitus, dorsal view **G** female habitus, ventral view. Abbreviations: CO = copulatory opening; DP = dorsal plate of the epigyne; FD = fertilization duct; S = spermatheca; VP = ventral plate of epigyne. Scale bars: equal for **F** and **G**.

Figures 12 , 13 , 16

#### Type material.

***Holotype***: ♂ (IZCAS-Ar40333), China, Tibet, Lhoka, Samyai Town, along the way to Qingpu Practice Cave, 29.38°N, 91.55°E, elevation ca. 4231 m, 15.VIII.2018, X. Zhang and J. Liu leg. ***Paratypes***: 1♂1♀ (IZCAS-Ar40334-Ar40335), same data as holotype; 2♀ (IZCAS-Ar40336-Ar40337), Lhoka, Aza Town, along the way to Zonggongbu Cave, 29.37°N, 91.32°E, elevation ca. 4389 m, 14.VIII.2018, X. Zhang and J. Liu leg.

#### Etymology.

The specific name is a noun in apposition taken from the type locality.

#### Diagnosis.

The male of *Pimoa samyai* sp. nov. resembles *P. binchuanensis* (see Zhang and Li 2019: 3, figs 1, 2) and *P. crispa* Hormiga, 1994 (see Hormiga 1994a: 63, figs 233–238; Hormiga 1994b: fig. 1A, B) but can be distinguished by the short and distally narrow cymbial denticulate process (Fig. 12B, vs. long and distally wide in *P. binchuanensis*; vs. distally wide in *P. crispa*); distinguished from *P. binchuanensis* by the nearly L-shaped pimoid cymbial sclerite (Fig. 12B, vs. nearly U-shaped); distinguished from *P. crispa* by a palpal tibia that is ca. 1/2 of the cymbial length (Fig. 12A–C, vs. tibia almost the same length as cymbium). The female of *P. samyai* resembles *P. crispa* Hormiga, 1994 (see Hormiga 1994a: 63, figs 239–247) and *P. indiscreta* Hormiga, 1994 (see Hormiga 1994a: 66, figs 248–255) but can be distinguished by the distance between the pair of spermathecae which is ca. 1/2 the width of a spermatheca (Fig. 13C, vs. separated by ca. 1/4 the width of a spermatheca in *P. crispa* and *P. indiscreta*) and by the distally wide dorsal plate (Fig. 13C, vs. distally narrow in *P. crispa* and *P. indiscreta*).

#### Description.

**Male** (**holotype**): Total length 6.92. Carapace 3.33 long, 2.63 wide. Abdomen 3.59 long, 2.31 wide. Eye sizes and interdistances: AME 0.16, ALE 0.17, PME 0.15, PLE 0.16; AME-AME 0.14, AME-ALE 0.14, PME-PME 0.16, PME-PLE 0.19. Leg measurements: I: 31.92 (8.85, 10.06, 9.74, 3.27); II: 31.40 (8.33, 9.94, 9.99, 3.14); III: 19.81 (5.83, 6.03, 5.96, 1.99); IV: 25.77 (7.31, 8.27, 7.88, 2.31). Habitus as in Fig. 13E. Carapace yellowish with black lateral margins; thoracic fovea and radial grooves distinct; sternum brownish. Abdomen black with yellowish transverse chevron bands. Legs brownish with black annulations, especially distinct on legs III and IV. Palp (Fig. 12A–C): patella short, ca. 1/2 of tibial length, with one retrolateral macroseta; tibia long, ca. 1/2 of cymbial length, with several macrosetae and a dorsal process; paracymbium short, ca. 1/3 of cymbial length, hook-shaped; pimoid cymbial sclerite nearly L-shaped, ca. 1/2 of cymbial length; cymbial denticulate process short and distally narrow, with more than ten cuspules; median apophysis indistinct; conductor distinct; pimoid embolic process pointed distally, longer than embolus; embolus beginning at the 5:30 o’clock position; embolic tooth absent.

**Female** (**paratype**): Total length 10.51. Carapace 4.81 long, 3.46 wide. Abdomen 5.70 long, 4.42 wide. Eye sizes and interdistances: AME 0.20, ALE 0.20, PME 0.19, PLE 0.19; AME-AME 0.16, AME-ALE 0.17, PME-PME 0.17, PME-PLE 0.23. Leg measurements: I: 31.73 (8.91, 10.51, 9.04, 3.27); II: 28.59 (7.95, 9.49, 8.14, 3.01); III: 20.19 (5.83, 6.54, 5.83, 1.99); IV: 26.85 (7.88, 8.97, 7.50, 2.50). Habitus as in Fig. 13F, G. Carapace yellowish with black lateral margins; thoracic fovea and radial grooves distinct; sternum brownish. Abdomen yellowish with black parts. Legs brownish with distinct black annulations on all legs. Epigyne (Fig. 13A–D): triangular; ventral plate broad, length subequal to width; dorsal plate longer than wide, nearly tongue-shaped; copulatory openings distinct; spermathecae nearly oval, separated by ca. 1/2 width of spermatheca; fertilization ducts anteriorly oriented.

#### Distribution.

Known only from the type locality, Tibet, China (Fig. 16).

### Pimoa yadong

Taxon classificationAnimaliaAraneaePimoidae

Zhang & Li, sp. nov .

E3550658-A604-5F0D-B776-921DAB9AF950

http://zoobank.org/4ED45938-95D8-43C3-B000-AAC071D0258E

**Figure 14.**
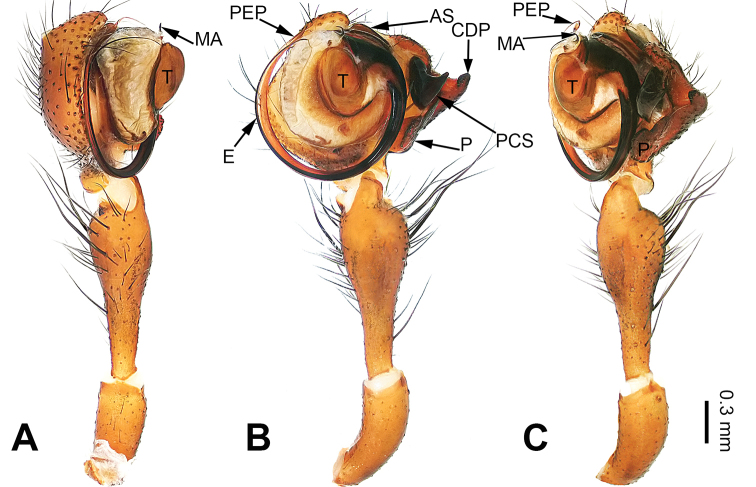
Left palp of *Pimoa yadong* sp. nov., holotype **A** prolateral view **B** ventral view **C** retrolateral view. Abbreviations: AS = alveolar sclerite; CDP = cymbial denticulate process; E = embolus; MA = median apophysis; P = paracymbium; PCS = pimoid cymbial sclerite; PEP = pimoid embolic process; T = tegulum. Scale bar: equal for **A–C**.

**Figure 15.**
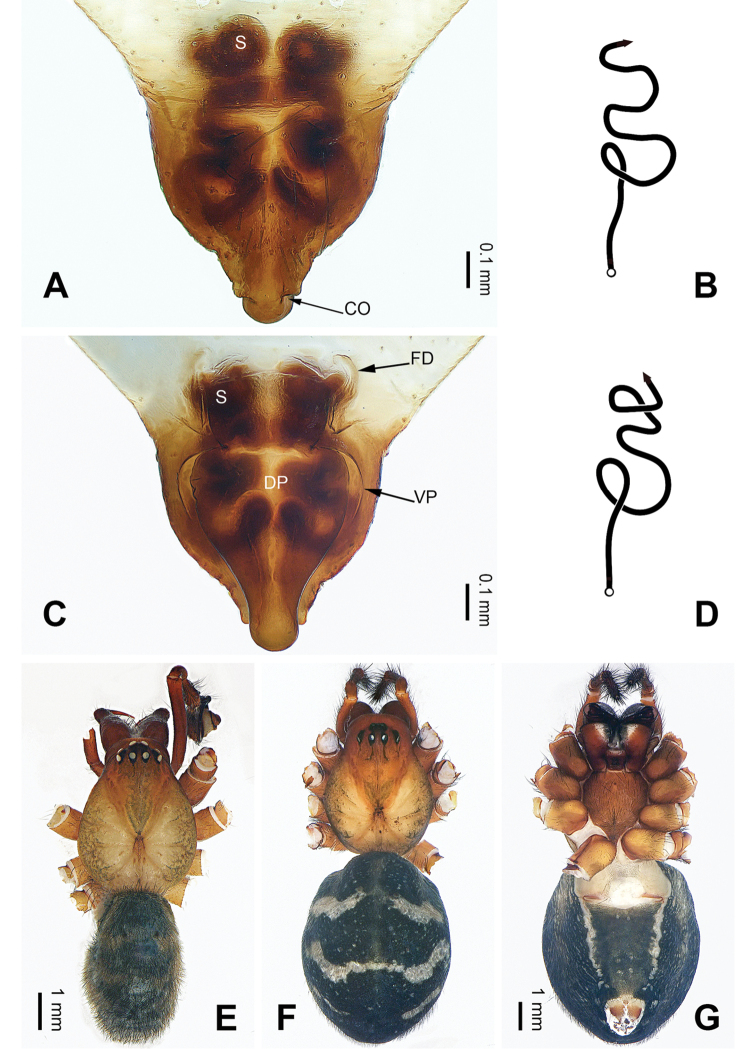
Epigyne and habitus of *Pimoa yadong* sp. nov., female paratype and male holotype **A** epigyne, ventral view **B** schematic course of internal duct system, ventral view **C** vulva, dorsal view **D** schematic course of internal duct system, dorsal view **E** male habitus, dorsal view **F** female habitus, dorsal view **G** female habitus, ventral view. Abbreviations: CO = copulatory opening; DP = dorsal plate of the epigyne; FD = fertilization duct; S = spermatheca; VP = ventral plate of epigyne. Scale bars: equal for **F** and **G**.

**Figure 16.**
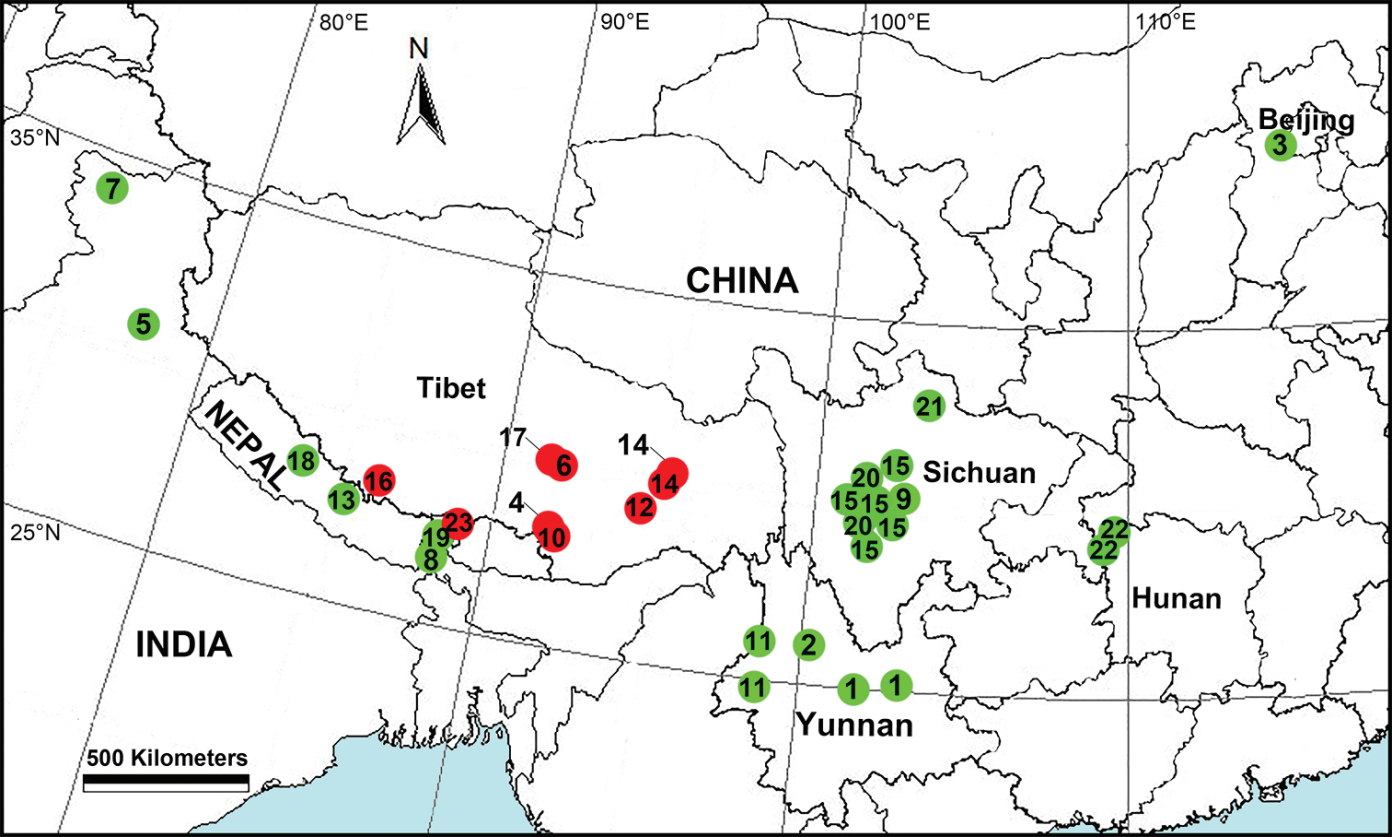
Distribution of *Pimoa* species from Asia. Red dots represent new species, green dots represent previously described species. **1***P. anatolica***2***P. binchuanensis***3***P. clavata***4***P. cona* sp. nov. **5***P. crispa***6***P. duiba* sp. nov. **7***P. gandhii***8***P. indiscreta***9***P. lata***10***P. lemenba* sp. nov. **11***P. lihengae***12***P. mainling* sp. nov. **13***P. nematoides***14***P. nyingchi* sp. nov. **15***P. reniformis***16***P. rongxar* sp. nov. **17***P. samyai* sp. nov. **18***P. sinuosa***19***P. thaleri***20***P. trifurcata***21***P. wanglangensis***22***P. xinjianensis***23***P. yadong* sp. nov.

Figures 14 , 15 , 16

#### Type material.

***Holotype***: ♂ (IZCAS-Ar40338), China, Tibet, Shigatse, Yadong County, along the way to the Qing Dynasty Customs Site, 27.42°N, 88.92°E, elevation ca. 2953 m, 6.VIII.2018, X. Zhang and J. Liu leg. ***Paratype***: 1♀ (IZCAS-Ar40339), same data as holotype.

#### Etymology.

The specific name is a noun in apposition taken from the type locality.

#### Diagnosis.

The male of *Pimoa yadong* sp. nov. resembles *P. nematoides* Hormiga, 1994 (see Hormiga 1994a: 71, figs 285–289) and *P. sinuosa* Hormiga, 1994 (see Hormiga 1994a: 67, figs 256–265) but can be distinguished by the wide and subtriangular pimoid cymbial sclerite (Fig. 14B, vs. proximally wide, distally narrow and curved in *P. nematoides*; vs. slender and distally blunt and curved in *P. sinuosa*), by the long palpal tibia, ca. 2 times longer than the cymbium (Fig. 14A–C, vs. short tibia, ca. 1/3 of cymbial length in *P. nematoides* and *P. sinuosa*). The female of *P. yadong* resembles *P. sinuosa* (see Hormiga 1994a: 67, figs 266–284) and *P. cona* sp. nov. (Fig. 2) but can be distinguished by a pair of nearly round spermathecae that are almost touching one another (Fig. 15A, vs. elliptic and separated spermathecae in *P. cona*; vs. subtriangular and separated spermathecae in *P. sinuosa*) and by the dorsal plate which extends beyond the ventral plate (Fig. 15C, vs. a dorsal plate shorter than the ventral plate in *P. nematoides* and *P. sinuosa*).

#### Description.

**Male** (**holotype**): Total length 8.46. Carapace 4.04 long, 3.27 wide. Abdomen 4.42 long, 2.95 wide. Eye sizes and interdistances: AME 0.21, ALE 0.19, PME 0.18, PLE 0.20; AME-AME 0.13, AME-ALE 0.18, PME-PME 0.17, PME-PLE 0.19. Leg measurements: I: – (11.60, –, –, –); II: – (10.13, –, –, –); III: 24.42 (7.18, 7.56, 7.18, 2.50); IV: – (8.91, 9.94, 9.04, –). Habitus as in Fig. 15E. Carapace yellowish with black lateral margins; thoracic fovea and radial grooves distinct; sternum brownish. Abdomen black with slightly yellowish transverse bands. Legs brownish with black annulations, especially distinct on legs III and IV. Palp (Fig. 14A–C): patella long, ca. 1/2 of tibial length; tibia long, ca. 2 times longer than cymbium, with several macrosetae and a dorsal process; paracymbium short, ca. 1/3 of cymbial length; pimoid cymbial sclerite wide and subtriangular, ca. 1/3 of cymbial length; cymbial denticulate process short and distally pointed, with more than five cuspules; median apophysis slender; conductor indistinct; pimoid embolic process pointed distally, almost the same length as embolus; embolus beginning at the 2:00 o’clock position; embolic tooth absent.

**Female** (**paratype**): Total length 11.86. Carapace 4.55 long, 3.85 wide. Abdomen 7.31 long, 5.38 wide. Eye sizes and interdistances: AME 0.19, ALE 0.22, PME 0.21, PLE 0.22; AME-AME 0.19, AME-ALE 0.26, PME-PME 0.24, PME-PLE 0.28. Leg measurements: I: 43.08 (11.60, 14.04, 12.82, 4.62); II: 34.30 (10.58, 8.91, 11.09, 3.72); III: 24.30 (7.44, 7.69, 6.86, 2.31); IV: – (9.42, –, –, –). Habitus as in Fig. 15F–G. Carapace yellowish with black lateral margins; thoracic fovea and radial grooves distinct; sternum brownish. Abdomen black with yellowish transverse chevron bands and vertical band not extending to the distal part. Legs brownish with black annulations, especially distinct on legs III and IV. Epigyne (Fig. 15A–D): subtriangular; ventral plate broad, length subequal to width; dorsal plate narrowing distally, extending beyond the ventral plate; copulatory openings distinct; spermathecae round, close to each other; fertilization ducts laterally oriented.

#### Distribution.

Known only from the type locality, Tibet, China (Fig. 16).

## Discussion

As a relict group, pimoids are ideal organisms for biogeographic study (Wang et al. 2008). Wang et al. (2008) estimated the divergence time of the North American and Asian species of *Pimoa* was approximately 110 Ma, and suggested that the discontinuous distribution was probably a consequence of the break-up of Laurasia. Mammola et al. (2016) inferred that European pimoids probably originated in the alpine region as a result of range contractions following dramatic climatic changes in the Alps after the mid Miocene.

Based on our spider collections in the last years, we have found that many *Pimoa* species have colonized in the southern region of the Tibetan Plateau. This study describes eight new species, yielding a total of 17 *Pimoa* species from China. However, this is only the tip of the iceberg of Chinese *Pimoa* species, and more new species will be reported with further collections. Phylogeographic analysis of Pimoidae from China will be conducted when the majority of *Pimoa* species appear to be recorded.

## Supplementary Material

XML Treatment for Pimoa

XML Treatment for Pimoa cona

XML Treatment for Pimoa duiba

XML Treatment for Pimoa lemenba

XML Treatment for Pimoa mainling

XML Treatment for Pimoa nyingchi

XML Treatment for Pimoa rongxar

XML Treatment for Pimoa samyai

XML Treatment for Pimoa yadong
